# Clinical Characteristics and Pharmacokinetics Change of Long-Term Responders to Antiprogrammed Cell Death Protein 1 Inhibitor Among Patients With Advanced NSCLC

**DOI:** 10.1016/j.jtocrr.2023.100474

**Published:** 2023-02-11

**Authors:** Hitomi Jo, Tatsuya Yoshida, Shigehiro Yagishita, Mayu Ohuchi, Yuji Matsumoto, Yuki Shinno, Yusuke Okuma, Yasushi Goto, Hidehito Horinouchi, Noboru Yamamoto, Kazuhisa Takahashi, Noriko Motoi, Akinobu Hamada, Yuichiro Ohe

**Affiliations:** a Department of Thoracic Oncology, National Cancer Center Hospital, Tokyo, Japan; b Division of Molecular Pharmacology, National Cancer Center Research Institute, Tokyo, Japan; c Department of Respiratory Medicine, Juntendo University Graduate School of Medicine, Tokyo, Japan; d Department of Experimental Therapeutics, National Cancer Center Hospital, Tokyo, Japan; e Department of Endoscopy, Respiratory Endoscopy Division, National Cancer Center Hospital, Tokyo, Japan; f Department of Diagnostic Pathology, National Cancer Center Hospital, Tokyo, Japan; g Division of Genome Biology, National Cancer Center Research Institute, Tokyo, Japan

**Keywords:** Anti–PD-1 inhibitor, Non–small cell lung cancer, Long-term response, Pharmacokinetics

## Abstract

**Introduction:**

Immune checkpoint inhibitors (ICIs) induce long-term, durable responses in patients with advanced NSCLC. Nevertheless, these responses are limited to a few patients, and most responders have disease progression. The purpose of this study was to determine the differences in clinical factors and blood drug concentrations between long-term responders (LTRs) and non-LTRs.

**Methods:**

We retrospectively analyzed consecutive patients with advanced NSCLC who received antiprogrammed cell death protein 1 (PD-1) inhibitor monotherapy (nivolumab) from December 22, 2015, to May 31, 2017. Patients who obtained a clinical benefit for more than 6 months were referred to as “responders”; among these, individuals who had a durable response for more than 2 years were defined as “LTRs.” Those with a clinical benefit for less than 2 years were defined as “non-LTRs.”

**Results:**

A total of 212 patients received anti–PD-1 inhibitor monotherapy. The responders accounted for 35% (75 of 212) of the patients. Of these, 29 (39%) were LTRs and 46 (61%) were non-LTRs. The overall response rate and median tumor shrinkage in the LTR group were significantly higher than those in the non-LTR group (76% versus 35%, *p* < 0.0001, and 66% versus 16%, *p* < 0.001, respectively). The groups had no significant difference in PD-L1 expression and serum drug concentration at 3- and 6-month post-treatment initiation.

**Conclusions:**

Significant tumor shrinkage was associated with a long-term response to an anti–PD-1 inhibitor. Nevertheless, the PD-L1 expression level and pharmacokinetic profile of the inhibitor could not be used to predict the durable response among the responders.

## Introduction

Immune checkpoint inhibitors (ICIs), such as programmed cell death protein 1 (PD-1) or programmed death-ligand 1 (PD-L1) inhibitors, have become the standard of treatment for patients with advanced NSCLC because they have better clinical outcomes and possess favorable safety profiles compared with chemotherapy.^1^, ^2^, ^3^, ^4^, ^5^, ^6^ ICIs induce durable clinical responses; however, these are usually limited to a few patients receiving the treatment. Most responders continue to have disease progression even after a long-lasting response.

PD-L1 expression in tumor cells is a prognosis marker for anti–PD-1 or PD-L1 therapy in advanced NSCLC. Patients with NSCLC with high PD-L1 expression (≥50%) treated with pembrolizumab exhibited longer survival times than those treated with platinum doublet chemotherapy. The KEYNOTE-024 study revealed that the response rate of patients with advanced NSCLC exhibiting high PD-L1 expression to pembrolizumab was 44.8%.^7^ In the KEYNOTE-042 study, evaluating the efficacy of pembrolizumab in treatment-naive patients with NSCLC with PD-L1 expression–positive tumor, the duration of response did not differ according to PD-L1 expression levels (PD-L1 tumor proportion score [TPS] 1%–49% versus ≥50%), whereas overall survival (OS) in patients with PD-L1 high expression was better compared with those with PD-L1 low expression.^8^ Consequently, there is an urgent need to determine the association between the clinical factors in patients with NSCLC and response duration.

Pharmacokinetics (PK) is one of the candidates for predicting the clinical factors associated with the response to treatment with ICI. Basak et al.^9^ previously revealed that patients with higher trough concentrations of nivolumab in their blood exhibited better treatment outcomes and longer survival. In patients with advanced NSCLC treated with pembrolizumab, the rapid clearance of pembrolizumab was strongly associated with poor prognosis.^10^ These data suggest that the blood concentration of ICI may be associated with treatment efficacy and survival; however, data on patients receiving long-term ICI are limited.^11^^,^^12^

The aim of this study was to characterize the clinical factors and the blood drug concentrations of patients who exhibited a long-term response (>2 y).

## Material and Methods

### Patients

We retrospectively analyzed consecutive patients with advanced NSCLC who received anti–PD-1 inhibitor monotherapy (nivolumab) at the National Cancer Center Hospital (Tokyo, Japan) from December 22, 2015, to May 31, 2017. This study was approved by the National Cancer Center Institutional Review Board (2015-355). Patients with no measurable lesions or response evaluations were excluded. Data on pretreatment patient characteristics, including age, sex, Eastern Cooperative Oncology Group performance status (PS), and smoking history, were collected. Tumor characteristics were noted, including histology, tumor molecular profiling for EGFR and ALK, PD-L1 status, tumor-node-metastasis classification proposed by the Union for International Cancer Control, and the number of metastatic organs. Data on treatment characteristics, including the regimen and the number of treatment lines, were also collected. Albumin, lactate dehydrogenase, and C-reactive protein levels and neutrophil-to-lymphocyte ratio were measured before nivolumab administration. EGFR mutation was assessed on the basis of polymerase chain reaction-based methods (therascreen EGFR RGQ PCR Kit [Scorpion-ARMS technology], QIAGEN, Hilden, Germany, and Cobas EGFR Mutation Test v2; Roche Diagnostics, Basel, Switzerland). ALK gene rearrangement status was assessed by fluorescent in situ hybridization or immunohistochemistry. PD-L1 expression in tumor cells was assessed using the 22C3 pharm Dx assay (Agilent, Santa Clara, CA). Expression was categorized using TPS, which was defined as the percentage of tumor cells that manifest membranous staining for PD-L1.^13^ PD-L1 TPS of at least 1% was considered positive, whereas that of more than 50% was considered a high expression.

### Tumor Response to Anti–PD-1 Inhibitor

Tumor responses were classified according to the Response Evaluation Criteria for Solid Tumors version 1.1.^14^ Maximal tumor shrinkage (MTS) was defined as the greatest tumor shrinkage compared with the patient's baseline at any follow-up assessment. Progression-free survival (PFS) was defined as the time from the first day of nivolumab or pembrolizumab treatment to the detection of the earliest signs of disease progression by computed tomography or magnetic resonance imaging or death from any cause. Patients who discontinued anti–PD-1 inhibitors owing to adverse events were also included in the analysis. The cutoff date was January 1, 2020. We defined patients who obtained clinical benefit (complete response [CR], partial response [PR], and stable disease) for more than 6 mo as “responders” and patients who obtained a clinical benefit for less than 6 mo as “nonresponders” on the basis of results of previous studies.^5^^,^^15^, ^16^, ^17^, ^18^ The patients who had a durable response for more than 2 years were defined as “long-term responder” (LTR) and those who exhibited clinical benefits for less than 2 years were referred to as “non-LTR,” because most of the patients without disease progression at 2 years after treatment with anti–PD-1 inhibitor remained progression-free at 5 years and survived more than 5 years.^1^^,^^19^

### PK Analysis of Nivolumab

The serum nivolumab concentrations were analyzed before treatment and at 3, 6, 12, 24, and 36 months after treatment in the study subjects. In patients receiving 3 mg/kg of nivolumab every 2 weeks, the serum samples were collected 14 to 21 days after the last dose and immediately before the next dose of nivolumab. Patients who had discontinued nivolumab owing to immune-related adverse events were excluded from the analysis. Nivolumab signature peptide quantitation was performed using a liquid chromatography-electrospray ionization-mass spectrometer with a triple quadrupole (Nexera X2 and LCMS-8050; Shimadzu, Kyoto, Japan).^20^ Serial serum samples were collected from patients with advanced NSCLC treated with nivolumab at the National Cancer Center Hospital. These samples were registered in the NCC Biobank (Tokyo, Japan) and stored at −20°C until use. In this study, the serum samples were retrospectively obtained from the NCC Biobank.

### Statistical Analysis

The differences in patient characteristics between the LTR and non-LTR groups were analyzed using the chi-square test or Fisher’s exact test. The PFS was estimated using the Kaplan-Meier method. The serum nivolumab concentrations were compared between the two groups using an unpaired *t* test. A comparative analysis of serum nivolumab concentration in multiple groups was performed using an analysis of variance. Differences were considered statistically significant at a probability value of less than 0.05. Statistical analyses were performed using STATA version 15.0 (Stata Corp., College Station, TX) and GraphPad Prism version 9.0.0 (GraphPad Software, San Diego, CA).

## Results

### Patient Characteristics

**Figure 1:**
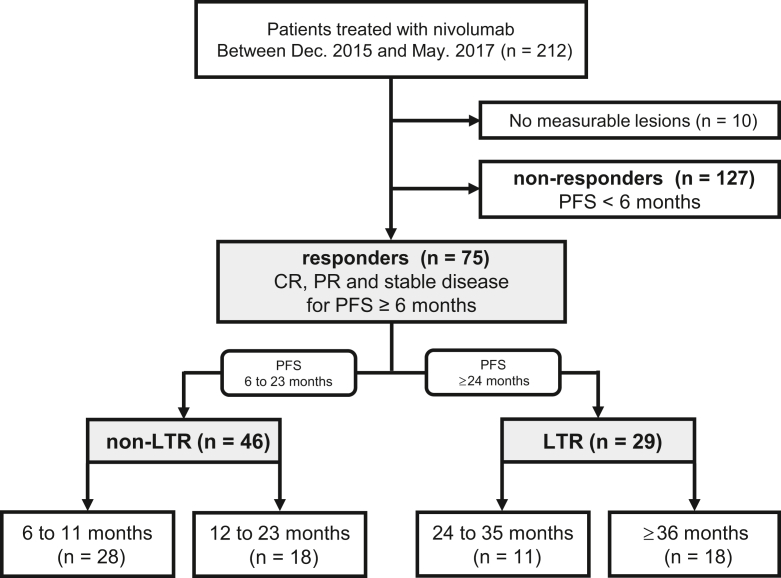
Flow diagram of the study. CR, complete response; Dec., December; LTR, long-term responder; PFS, progression-free survival; PR, partial response.

**Figure 2:**
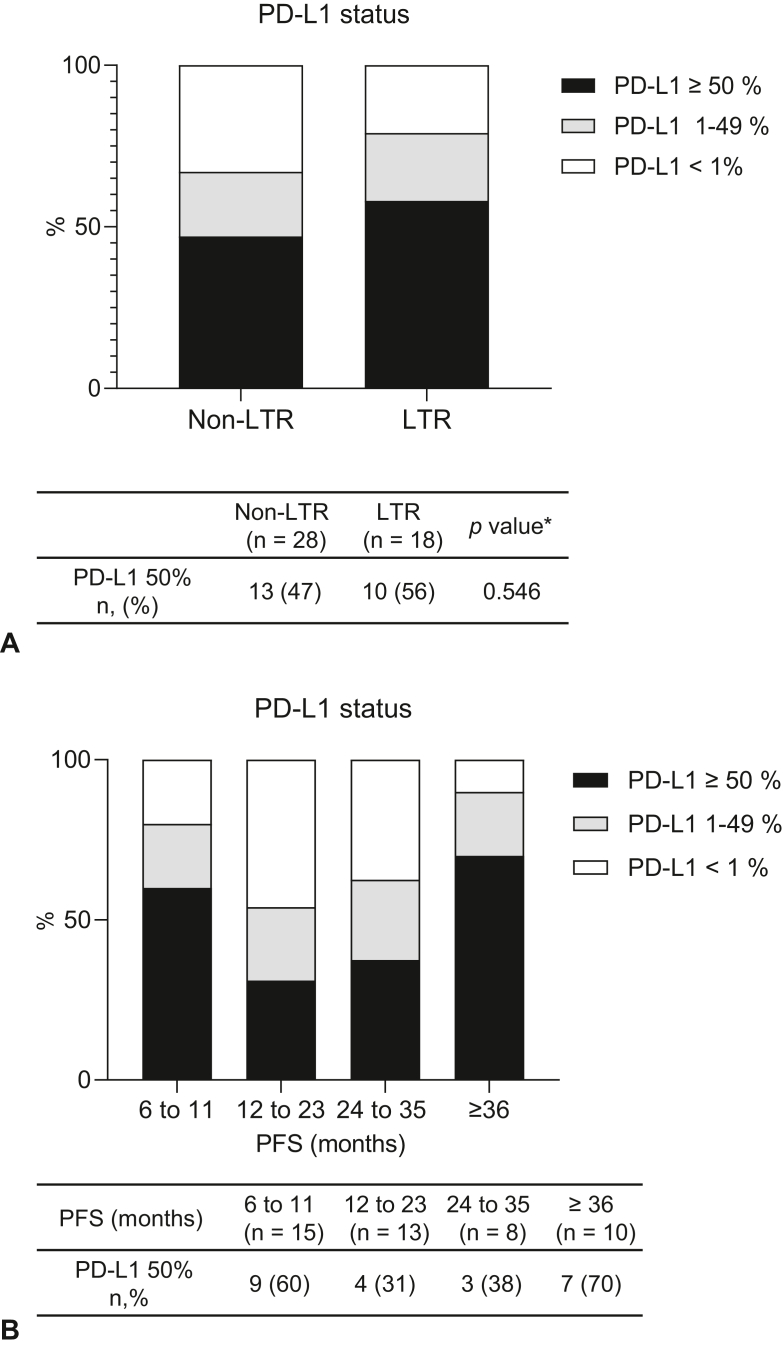
PD-L1 status in patients with advanced NSCLC. (*A*) Bar graph illustrating the PD-L1 status (PD-L1 ≥50%, PD-L1 1%–49%, and PD-L1 <1%) in the non-LTR (n = 28) and LTR groups (n = 18) of patients treated with anti–PD-1 inhibitors. ∗PD-L1 expression levels of greater than or equal to 50% and less than 50% were compared between the patients in the LTR and non-LTR groups. (*B*) Bar graph illustrating the PD-L1 status (PD-L1 ≥50%, PD-L1 1%–49%, and PD-L1 <1%) for each response period (6–11, 12–23, 24–35, and ≥36 mo) in patients treated with anti–PD-1 inhibitors. LTR, long-term responder; PD-1, programmed cell death protein 1; PD-L1, programmed death-ligand 1; PFS, progression-free survival.

We identified 212 consecutive patients who had received anti–PD-1 inhibitor monotherapy. Of these, 10 patients with no measurable lesions were excluded and 127 patients who had a response to ICI for less than 6 months were also excluded (non-responders). As a result, 75 (35%) patients were classified as responders, with a treatment effect of more than 6 months (Fig. 1). Among them, 29 (39%) belonged to the LTR group and 46 (61%) belonged to the non-LTR group. The median follow-up time was 39.7 months in the LTR group and 20.6 months in the non-LTR group. Median PFS was not reached (NR) (95% confidence interval [CI]: 46.52–NR) and 11.0 months (95% CI: 8.74–12.65), respectively (Supplementary Fig. 1*A* and *B*). The duration of clinical benefit was classified as follows: 6 to 11 months (n = 28), 12 to 23 months (n = 18), 24 to 35 months (n = 11), and more than or equal to 36 months (n = 18). All patients in this study were Japanese. Table 1 illustrates the baseline characteristics of the patients in the LTR and non-LTR groups. The median age was 65 (range: 33–83) years, 59 (79%) patients were male, 67 (89%) had a smoking history, and 71 (95%) had an Eastern Cooperative Oncology Group PS of 0 or 1. No significant differences were identified in the clinical factors between the LTR and non-LTR groups. Among the histologic subtypes, nonsquamous cell histology was more common in the LTR group (90% versus 67%, *p* = 0.028). All patients received nivolumab monotherapy (3 mg/kg or 240 mg/body, intravenously, every 2 wk). Next, we evaluated the association between the clinical characteristics. The number of metastatic organs and biological parameters, such as serum albumin, lactate dehydrogenase, and C-reactive protein levels and neutrophil-to-lymphocyte ratio, was not associated with response duration. Moreover, among the responders, high PD-L1 expression was not associated with LTR (Fig. 2*A* and *B*).

**Table 1:** Patients Baseline Characteristics

Characteristics	Non-LTR (n = 46)	LTR (n = 29)	*p* Value^a^
Age, median (range)	64 (37–83)	67 (42–79)	0.622
Sex			
Male	37 (80)	22 (76)	0.638
Female	9 (20)	7 (24)	
ECOG PS			
0–1	43 (93)	28 (97)	0.564
2–3	3 (7)	1 (3)	
Stage			
Recurrence	12 (26)	6 (21)	
III	14 (30)	10 (34)	
IV	20 (44)	13 (45)	
Smoking status			
Never	7 (15)	1 (3)	0.108
Current or former	39 (85)	28 (97)	
Histology			
Non-Sq	31 (67)	26 (90)	0.028
Sq	15 (33)	3 (10)	
Driver gene^b^			
Yes.	7 (15)	2 (6)	0.280
No.	39 (85)	27 (94)	
PD-L1 expression			
≥50%	11 (24)	10 (34)	
1%–49%	6 (13)	4 (14)	
<1%	9 (20)	4 (14)	
Unknown	20 (43)	11 (38)	
Prior lines of therapy			
1	25 (54)	19 (66)	0.339
≥2	21 (46)	10 (34)	
No. of metastatic organ			
<3	26 (57)	18 (62)	0.635
≥3	20 (43)	11 (38)	
ALB			
<3.5	14 (30)	5 (17)	0.201
≥3.5	32 (70)	24 (83)	
LDH			
<250	37 (80)	21 (72)	0.419
≥250	9 (20)	8 (28)	
CRP			
<1	22 (48)	19 (66)	0.255
≥1	24 (52)	10 (34)	
NLR			
<5	33 (72)	22 (76)	0.694
≥5	13 (28)	7 (24)	

ALB, albumin; CRP, C-reactive protein; ECOG PS, Eastern Cooperative Oncology Group performance score; LDH, lactate dehydrogenase level; NLR, neutrophil to-lymphocyte ratio; PD-L1, programmed cell death-ligand 1; Sq, squamous cell carcinoma.

a *p* values were calculated by comparing the LTR and non-LTR groups.

b Driver gene was tested for EGFR mutation and ALK gene fusion.

Regarding the details of nivolumab treatment, the median duration of nivolumab treatment was 32.7 months in the LTR group and 8.5 months in the non-LTR group (Table 2). In this study, 28 patients discontinued nivolumab during the course of treatment. Among them, 12 (41%) patients in the LTR group and 13 (28%) in the non-LTR group were discontinued owing to immune-related adverse events. In contrast, no patients discontinued at 2 years after the initiation of nivolumab.

**Table 2:** Details of Nivolumab Treatment

Details of Treatment	Non-LTR (n = 46)	LTR (n = 29)
Median treatment time, mo	8.5	32.7
Nivolumab administration		
Continuation	31 (68)	16 (55)
Patient outcome: progression/ongoing^a^	31/0^a^	3/13^a^
Discontinuation during the course of treatment	15 (32)	13 (45)
Patient outcome: progression/ongoing^a^	15/0^a^	4/9^a^
Due to toxicity	13 (28)	12 (41)
Patient’s request	1 (2)	0 (0)
Due to another treatment	1 (2)	1 (4)

LTR, long-term responder.

a Breakdown of treatment outcomes for patients who continued or discontinued nivolumab treatment, respectively.

### Association Between Tumor Shrinkage and Long-Term Response

**Figure 3:**
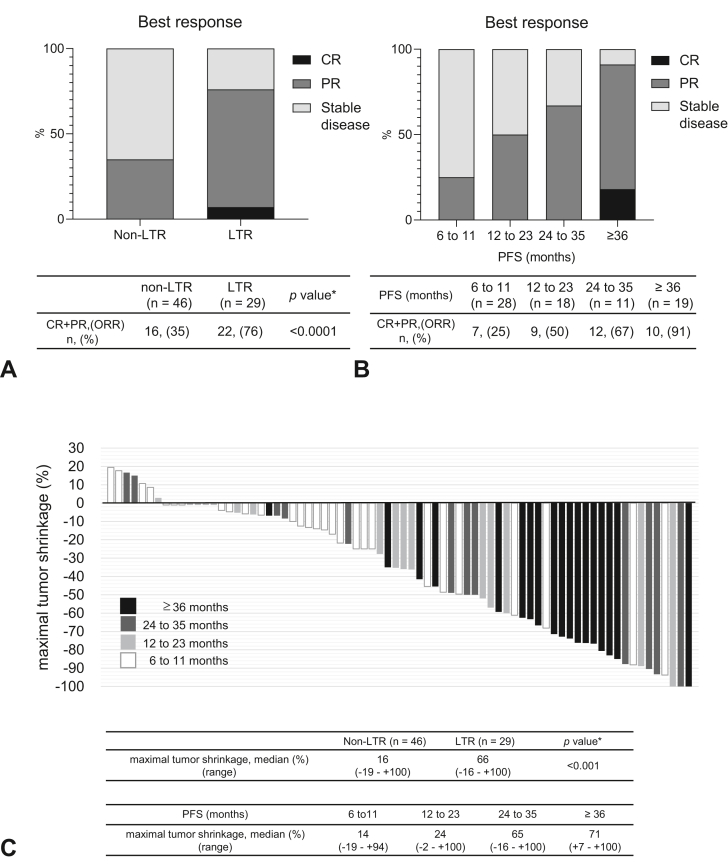
Best response and tumor shrinkage. (*A*) Bar graph illustrating the percentage of patients with the best response (CR, PR, and stable disease) to anti–PD-1 inhibitors in the non-LTR (n = 46) and LTR (n = 29) groups. (*B*) Bar graph illustrating the percentage of patients with the best response (CR, PR, and stable disease) to anti–PD-1 inhibitors at each response period (6–11, 12–23, 24–35, and ≥36 mo). (*C*) Waterfall plot of tumor shrinkage in the responders at each response period (6–11, 12–23, 24–35, and ≥36 mo). ∗*p* values were calculated by comparing the LTR and non-LTR groups. CR, complete response; LTR, long-term responder; ORR, objective response rate; PD-1, programmed cell death protein 1; PR, partial response.

The overall response rate (ORR) in the LTR group was significantly higher than that in the non-LTR group (76% versus 35%, *p* ＜ 0.0001; Fig. 3*A*). The ORR, classified by period, increased as the response duration lasted long: 25% (6–11 mo), 50% (12–23 mo), 67% (24–35 mo), and 91% (≥36 mo) (Fig. 3*B*). Figure 3*C* illustrates a waterfall plot of the tumor shrinkage in the target region. The median of MTS was 35% (range: −19% to +100%) in 75 responders. In each duration of response (6–11, 12–23, 24–35, and ≥36 mo), the median of the MTS was 14% (range: −19% to +94%), 24% (range: −2% to +100%), 65% (range: −16% to +100%), and 71% (range: +7% to +100%). The MTS in the LTR group was significantly higher than that in the non-LTR group (66% versus 16%, *p* < 0.001).

### PK in Patients Treated With Nivolumab

**Figure 4:**
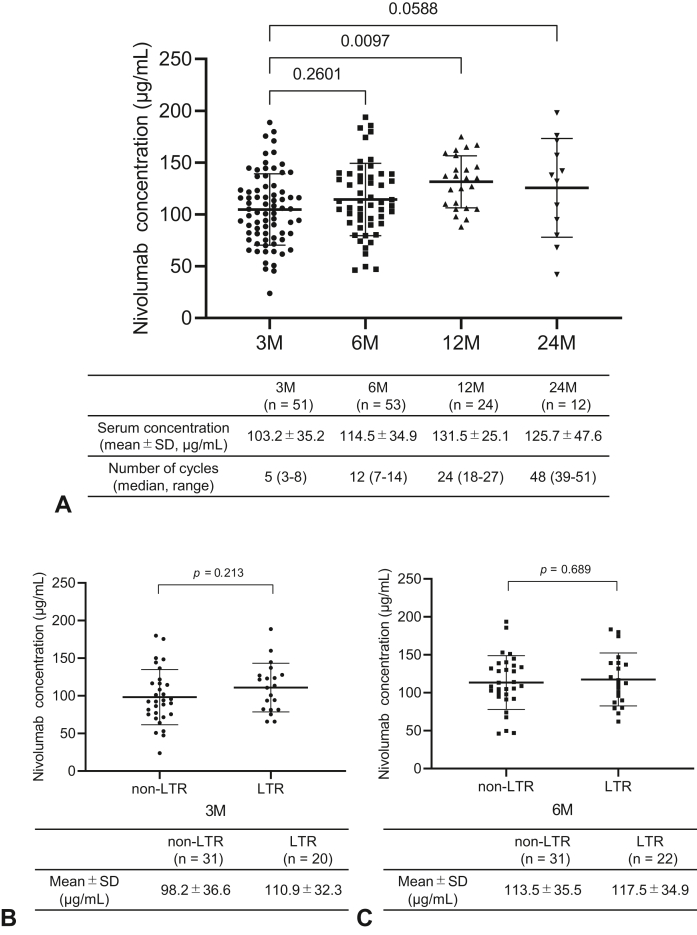
Pharmacokinetic analysis in patients treated with nivolumab. (*A*) Dot plot illustrating nivolumab trough concentrations at 3M, 6M, 12M, and 24M. (*B*) Dot plot illustrating nivolumab trough concentrations at 3M in the non-LTR (n = 31) and LTR (n = 20) groups. (*C*) Dot plot illustrating nivolumab trough concentrations at 6M in the non-LTR (n = 31) and LTR (n = 22) groups. ∗*p* values were calculated by comparing the 3M and 6M, the 3M and 12M, and the 3M and 24M groups. ∗∗*p* values were calculated by comparing the LTR and non-LTR groups. 3M, 3 months after the start of treatment; 6M, 6 months after the start of treatment; 12M, 12 months after the start of treatment; 24M, 24 months after the start of treatment; LTR, long-term responder.

A total of 254 serum samples from 74 patients who received nivolumab were used for PK analysis. The number of samples analyzed at 3, 6, 12, and 24 months after nivolumab administration was 51, 53, 24, and 12, respectively. After dosing, the serum nivolumab concentration gradually increased and reached a plateau stage at approximately 6 months postdrug administration (Fig. 4*A*) and did not differ between the LTR and non-LTR groups at 3 and 6 months (Fig. 4*B* and *C*). Serum nivolumab concentrations were not associated with the best response or PFS (Supplementary Fig. 2*A*–*C*).

## Discussion

In this study, we evaluated the clinical characteristics of patients with advanced NSCLC who had a durable response to anti–PD-1 inhibitor. Among the responders, the LTR group exhibited significantly greater tumor shrinkage than the non-LTR group, and the depth of the tumor response was associated with a durable response. Nevertheless, there was no difference in the proportion of patients with high PD-L1 expression between the LTR and non-LTR groups. PK analysis revealed that the nivolumab trough concentrations at 3 and 6 months after treatment initiation were not associated with a long-term response.

Pooled analysis from two phase 3 trials of nivolumab in previously treated patients with NSCLC (CheckMate-017 and -057) revealed that the 5-year OS and PFS rates were 13.4% and 8.0%, respectively.^1^ In a phase 3 trial evaluating the efficacy of pembrolizumab (KEYNOTE-010 trial), the 5-year OS and PFS rates were 25.0% and 18.2% in previously treated patients with NSCLC with PD-L1 TPS greater than or equal to 50% and were 15.6% and 9.4% in patients with PD-L1 TPS greater than or equal to 1%, respectively, suggesting a positive correlation between PD-L1 expression, PFS, and OS.^3^ Conversely, the duration of response did not differ according to the PD-L1 expression (PD-L1 TPS 1%–49% versus ≥50%).^21^ Our study revealed that a high PD-L1 expression was not associated with long-term response among the responders. In addition, high expression of PD-L1 was not associated with PFS in multivariate analysis including other factors (Supplementary Table 1).

We investigated and evaluated the clinical characteristics that could affect the response of LTRs to anti–PD-1 inhibitor. Previous reports have revealed that metastatic status, such as in the liver and central nervous system, and total tumor volume affected the clinical outcomes of anti–PD-1 therapy. In this study, we focused on the number of metastatic organs; however, the number of metastatic organs was not associated with LTRs in this study. Significant tumor shrinkage was associated with a long-term response to the anti–PD-1 inhibitor. McCoach et al.^22^ reported that the depth of tumor response was associated with longer PFS and OS in patients treated with ALK or anti–PD-1 inhibitors. Dimitriou et al.^23^ reported that the PFS rate at 5 years was higher in the CR and complete metabolic response groups than that in the PR/stable disease and noncomplete metabolic response groups at 1-year post-treatment initiation in patients treated with an anti–PD-1-based therapy having no tumor progression at 1 year. Therefore, the extent of tumor response, such as tumor shrinkage and metabolic response, among the responders can help predict the response duration in patients treated with ICIs. Nevertheless, further evaluation is needed to understand the use of the depth of tumor response in immunotherapeutic clinical trials.

Our study also investigated the association between nivolumab trough concentrations and durable response. Previous clinical trials have revealed that the dose of nivolumab and pembrolizumab did not influence their efficacy.^11^^,^^24^^,^^25^ Nevertheless, some reports indicate that exposure to ICIs and their clearance were associated with the therapeutic effect of anti–PD-L1 inhibitors.^12^^,^^26^ Basak et al.^9^ reported that the responders to nivolumab had higher mean trough concentrations after 2, 4, and 10 weeks of treatment compared with patients with disease progression and that the higher trough concentrations were associated with a longer OS. Turner et al.^10^ reported that higher pembrolizumab clearance paralleled with disease severity markers associated with end-stage cancer anorexia-cachexia syndrome and that the prognosis in such patients was significantly shorter than with those without it. In this study, the response duration did not correlate with serial nivolumab trough concentrations at 3- and 6-months post-treatment initiation. This discrepancy is due to the fact that the population in our study was focused on patients who had responded to treatment for more than 6 months.

This study has several limitations. First, this was a retrospective study, and the population size was small. Furthermore, the imaging frequency was at the physician’s discretion. Second, this study included patients treated with only nivolumab monotherapy, not pembrolizumab or ICIs in combination with chemotherapy. ICI in combination with chemotherapy has been the standard of care in patients with advanced NSCLC. It might be difficult to apply our findings to current clinical practice. Nevertheless, there have been few studies that focused on the clinical characteristics including PK parameters of LTRs to ICI. In addition, ICI is the main treatment to achieve the durable clinical benefit from the combination therapy, because the only chemotherapy represents limited efficacy in advanced NSCLC. Thus, the results from our study which focused on the clinical characteristics of LTRs to anti–PD-1 inhibitor will be meaningful. Third, our results did not reveal the predictive factors at baseline or the LTR because the PK change and the depth of response were determined after the ICI treatment. In contrast, the depth of the tumor shrinkage could be used as a surrogate for longer duration of response and help in deciding whether to discontinue ICI treatment. Fourth, this study lacked data on T-cell phenotypes, such as effector/memory cell and tumor-infiltrating lymphocyte (TIL) status within the tumor microenvironment (TME). Kim et al.^27^ reported that a low abundance of effector/memory cell subsets and a high abundance of CD8^+^ T lymphocytes in the peripheral blood were associated with worse prognosis in patients treated with anti–PD-1. We previously reported that the TIL status influences the clinical outcomes of anti–PD-1/PD-L1 therapy, even in patients with advanced NSCLC with tumors exhibiting high PD-L1 expression.^28^ Further investigation is needed on the association between the depth of tumor response and the T-cell phenotype and TIL status within the TME.

In conclusions, our study revealed that significant tumor shrinkage was associated with long-term response in patients benefiting from anti–PD-1 inhibitors. Nevertheless, PD-L1 expression and PK profiles could not be used to predict the production of a durable response among the responders.

## CRediT Authorship Contribution Statement

**Hitomi Jo**: Conceptualization, Methodology, Data curation, Formal analysis, Investigation, Visualization, Writing—original draft.

**Tatsuya Yoshida**: Conceptualization, Methodology, Resources, Data curation, Writing—original draft, Writing—review and editing.

**Shigehiro Yagishita**: Conceptualization, Methodology, Resources, Data curation, Writing—review and editing.

**Mayu Ohuchi**: Methodology, Validation, Investigation, Data curation, Writing—review and editing.

**Yuji Matsumoto:** Resources, Data curation, Writing—review and editing.

**Yuki Shinno**: Resources, Data curation, Writing—review and editing.

**Yusuke Okuma**: Resources, Data curation, Writing—review and editing.

**Yasushi Goto**: Resources, Data curation, Writing—review and editing.

**Hidehito Horinouchi**: Resources, Data curation, Writing—review and editing.

**Noboru Yamamoto**: Resources, Data curation, Writing—review and editing.

**Kazuhisa Takahashi**: Writing—review and editing.

**Noriko Motoi**: Resources, Data curation, Writing—review and editing.

**Akinobu Hamada**: Resources, Data curation, Writing—review and editing.

**Yuichiro Ohe**: Resources, Supervision, Writing—review and editing.

## References

[1] Borghaei H., Gettinger S., Vokes E.E. (2021). Five-year outcomes from the randomized, Phase III trials CheckMate 017 and 057: nivolumab versus docetaxel in previously treated non-small-cell lung cancer. J Clin Oncol.

[2] Gandhi L., Rodriguez-Abreu D., Gadgeel S. (2018). Pembrolizumab plus chemotherapy in metastatic non-small-cell lung cancer. N Engl J Med.

[3] Herbst R.S., Garon E.B., Kim D.W. (2021). Five year survival update from KEYNOTE-010: pembrolizumab versus docetaxel for previously treated, programmed death-ligand 1-positive advanced NSCLC. J Thorac Oncol.

[4] Reck M., Rodriguez-Abreu D., Robinson A.G. (2019). Updated analysis of KEYNOTE-024: pembrolizumab versus platinum-based chemotherapy for advanced non-small-cell lung cancer with PD-L1 tumor proportion score of 50% or greater. J Clin Oncol.

[5] Rittmeyer A., Barlesi F., Waterkamp D. (2017). Atezolizumab versus docetaxel in patients with previously treated non-small-cell lung cancer (OAK): a phase 3, open-label, multicentre randomised controlled trial. Lancet.

[6] Socinski M.A., Jotte R.M., Cappuzzo F. (2018). Atezolizumab for first-line treatment of metastatic nonsquamous NSCLC. N Engl J Med.

[7] Reck M., Rodriguez-Abreu D., Robinson A.G. (2016). Pembrolizumab versus Chemotherapy for PD-L1-Positive non-small-Cell Lung Cancer. N Engl J Med.

[8] Mok T.S.K., Wu Y.-L., Kudaba I. (2019). Pembrolizumab versus chemotherapy for previously untreated, PD-L1-expressing, locally advanced or metastatic non-small-cell lung cancer (KEYNOTE-042): a randomised, open-label, controlled, phase 3 trial. Lancet.

[9] Basak E.A., Koolen S.L.W., Hurkmans D.P. (2019). Correlation between nivolumab exposure and treatment outcomes in non-small-cell lung cancer. Eur J Cancer.

[10] Turner D.C., Kondic A.G., Anderson K.M. (2018). Pembrolizumab exposure-response assessments challenged by association of cancer cachexia and catabolic clearance. Clin Cancer Res.

[11] Centanni M., Moes D., Troconiz I.F., Ciccolini J., van Hasselt J.G.C. (2019). Clinical pharmacokinetics and pharmacodynamics of immune checkpoint inhibitors. Clin Pharmacokinet.

[12] Desnoyer A., Broutin S., Delahousse J. (2020). Pharmacokinetic/pharmacodynamic relationship of therapeutic monoclonal antibodies used in oncology: Part 2, immune checkpoint inhibitor antibodies. Eur J Cancer.

[13] Roach C., Zhang N., Corigliano E., Ponto G. (2016). Development of a companion diagnostic PD-L1 immunohistochemistry assay for pembrolizumab therapy in non-small-cell lung cancer. Appl Immunohistochem Mol Morphol.

[14] Eisenhauer E.A., Therasse P., Bogaerts J. (2009). New response evaluation criteria in solid tumours: revised RECIST guideline (version 1.1). Eur J Cancer.

[15] Brahmer J., Reckamp K.L., Baas P. (2015). Nivolumab versus docetaxel in Advanced squamous-Cell non-small-Cell Lung Cancer. N Engl J Med.

[16] Garon E.B., Rizvi N.A., Hui R. (2015). Pembrolizumab for the treatment of non-small-cell lung cancer. N Engl J Med.

[17] Rizvi N.A., Hellmann M.D., Snyder A. (2015). Cancer immunology. Mutational landscape determines sensitivity to PD-1 blockade in non-small cell lung cancer. Science.

[18] Shirasawa M., Yoshida T., Takayanagi D. (2021). Activity and immune correlates of programmed Death-1 blockade therapy in patients with advanced large cell neuroendocrine carcinoma. Clin Lung Cancer.

[19] Garon E.B., Hellmann M.D., Rizvi N.A. (2019). Five-year overall survival for patients with advanced nonsmall-cell lung cancer treated with pembrolizumab: results from the Phase I KEYNOTE-001 study. J Clin Oncol.

[20] Ohuchi M., Yagishita S., Taguchi K. (2021). Use of an alternative signature peptide during development of a LC-MS/MS assay of plasma nivolumab levels applicable for multiple species. J Chromatogr B Analyt Technol Biomed Life Sci.

[21] Reck M., Rodriguez-Abreu D., Robinson A.G. (2021). Five-year outcomes with pembrolizumab versus chemotherapy for metastatic non-small-cell lung cancer with PD-L1 tumor proportion score ≥ 50. J Clin Oncol.

[22] McCoach C.E., Blumenthal G.M., Zhang L. (2017). Exploratory analysis of the association of depth of response and survival in patients with metastatic non-small-cell lung cancer treated with a targeted therapy or immunotherapy. Ann Oncol.

[23] Dimitriou F., Lo S.N., Tan A.C. (2022). FDG-PET to predict long-term outcome from anti-PD-1 therapy in metastatic melanoma. Ann Oncol.

[24] Agrawal S., Feng Y., Roy A., Kollia G., Lestini B. (2016). Nivolumab dose selection: challenges, opportunities, and lessons learned for cancer immunotherapy. J Immunother Cancer.

[25] Freshwater T., Kondic A., Ahamadi M. (2017). Evaluation of dosing strategy for pembrolizumab for oncology indications. J Immunother Cancer.

[26] Feng Y., Wang X., Bajaj G. (2017). Nivolumab exposure-response analyses of efficacy and safety in previously treated squamous or nonsquamous non-small cell lung cancer. Clin Cancer Res.

[27] Kim C.G., Kim K.H., Pyo K.H. (2019). Hyperprogressive disease during PD-1/PD-L1 blockade in patients with non-small-cell lung cancer. Ann Oncol.

[28] Shirasawa M., Yoshida T., Shimoda Y. (2021). Differential immune-related microenvironment determines programmed cell death Protein-1/Programmed death-ligand 1 blockade efficacy in patients with advanced NSCLC. J Thorac Oncol.

