# Supplementary material for: Clinical Characteristics and Pharmacokinetics Change of Long-Term Responders to Antiprogrammed Cell Death Protein 1 Inhibitor Among Patients With Advanced NSCLC
Source: JTO Clin Res Rep. 2023 Feb 11;4(4):100474. doi: 10.1016/j.jtocrr.2023.100474 (PMC10050777; doi:10.1016/j.jtocrr.2023.100474)
Supplement: Supplemental Material [file mmc1.pptx]

## Slide 1
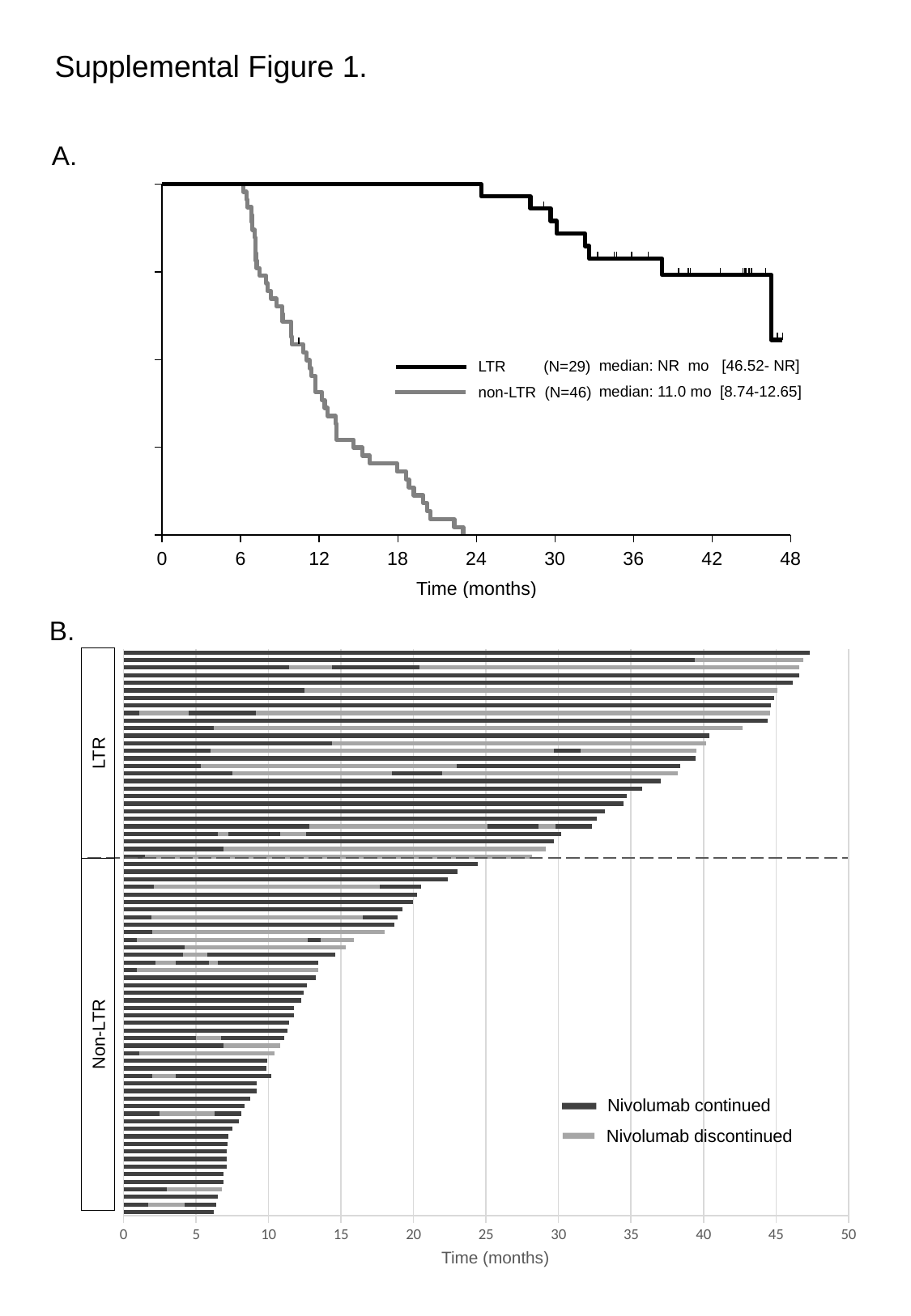

Supplemental Figure 1.
A.
median: NR mo [46.52- NR]
median: 11.0 mo [8.74-12.65]
B.
### Chart
| Category | PFS1 | 中止 | PFS２ | 中止 | PFS3 |
|---|---|---|---|---|---|LTR
Non-LTR
Nivolumab continued
Nivolumab discontinued
Time (months)

## Slide 2
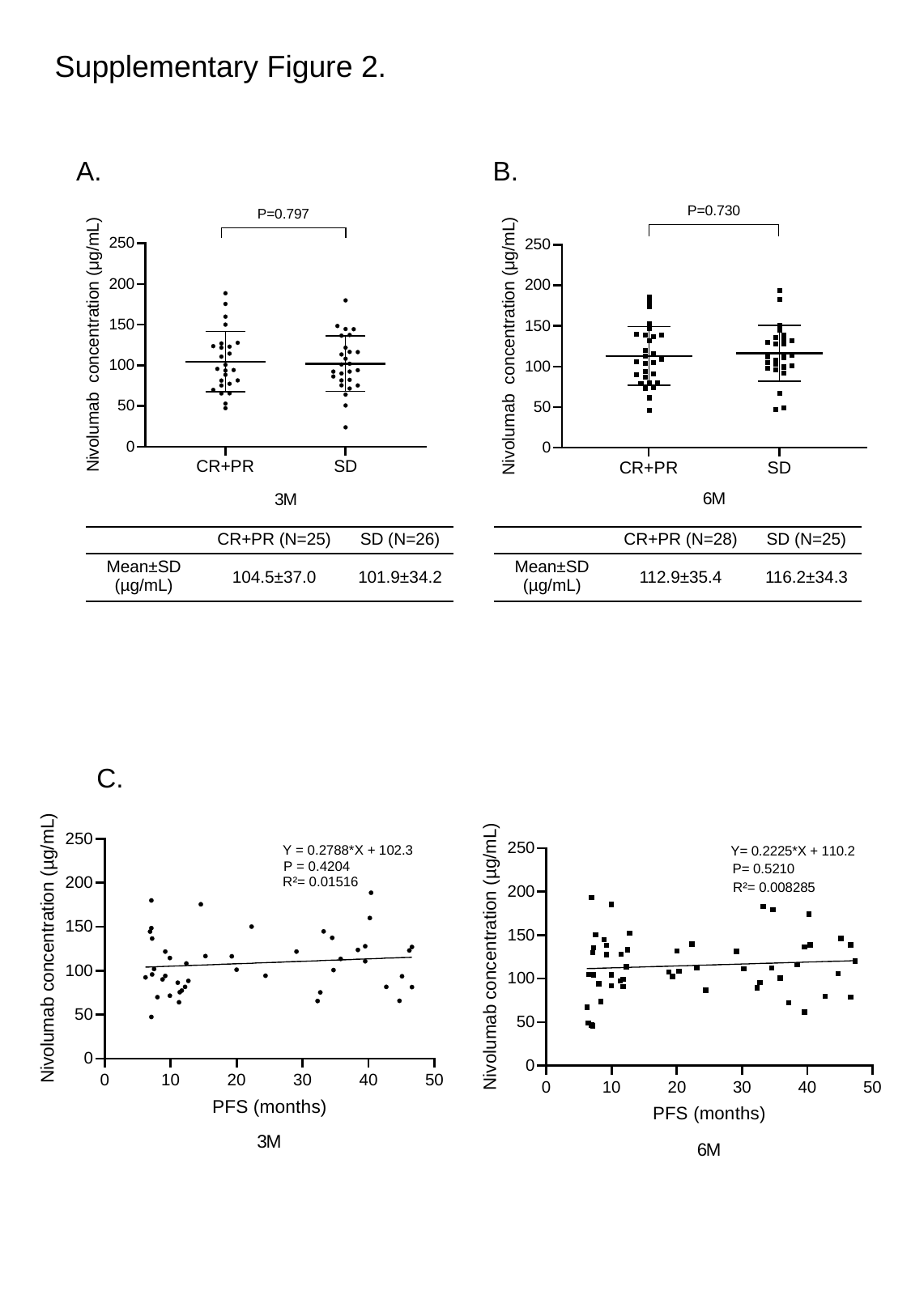

Supplementary Figure 2.
A.
B.
| | CR+PR (N=25) | SD (N=26) |
| --- | --- | --- |
| Mean±SD (µg/mL) | 104.5±37.0 | 101.9±34.2 |
| | CR+PR (N=28) | SD (N=25) |
| --- | --- | --- |
| Mean±SD (µg/mL) | 112.9±35.4 | 116.2±34.3 |
C.
